# AI-induced job crafting: a systematic review of cognitive appraisal pathways

**DOI:** 10.3389/fpsyg.2026.1788385

**Published:** 2026-03-31

**Authors:** Zhongrui Qiao

**Affiliations:** Department of Labour and Engineering Psychology, Lomonosov Moscow State University, Moscow, Russia

**Keywords:** AI integration, approach-avoidance behavior, artificial intelligence awareness, challenge-hindrance appraisal, cognitive appraisal, job crafting, systematic review

## Abstract

**Background:**

The integration of artificial intelligence (AI) into workplaces is increasingly associated with changes in job design and with employees’ efforts to adapt their work through job crafting. Evidence from the 15 included studies suggests that AI-related perceptions and cognitions, often broadly labeled as AI awareness in the literature, are not consistently directly associated with job crafting. Instead, the reviewed evidence suggests that their associations with job crafting are more consistently observed in conjunction with dual cognitive appraisal pathways. Challenge appraisals, in which AI is interpreted as an opportunity, are consistently linked to approach-oriented job crafting through mechanisms such as creative process engagement and harmonious work passion, whereas threat appraisals are associated with avoidance-oriented crafting through job insecurity and obsessive work passion. These patterns appear to vary depending on individual factors, such as AI-related knowledge and a positive stress mindset, as well as organizational conditions, including servant leadership and corporate social responsibility. Consequently, AI-related perceptions and cognitions cannot be regarded as uniformly beneficial or harmful; rather, their associations with job crafting depend on employees’ appraisals, the specific ways these constructs are operationalized, and the surrounding organizational context.

**Objective:**

This systematic review aims to elucidate the cognitive appraisal mechanisms through which heterogeneous AI-related perceptions and cognitions, broadly grouped under the label of AI awareness in the literature, are associated with employee job crafting behaviors.

**Methods:**

A comprehensive literature search was conducted in accordance with PRISMA guidelines across several major academic databases, including Web of Science, Scopus, PubMed, and PsycINFO. Empirical studies investigating the relationship between AI-related perceptions and cognitions, including AI exposure, perceived AI threat or opportunity, and AI-related knowledge, and job crafting among employee populations were included. Data were extracted and synthesized narratively to identify mediating mechanisms and potential moderating factors.

**Results:**

Evidence from the reviewed studies indicates that the various AI-related constructs broadly grouped under the label of AI awareness do not demonstrate consistent direct associations with job crafting. Instead, the relationships between these heterogeneous AI-related constructs and job crafting appear to operate through dual cognitive appraisal pathways. Challenge appraisals, in which AI is interpreted as an opportunity, are consistently linked to approach-oriented job crafting, often accompanied by mechanisms such as engagement in creative processes and harmonious work passion. In contrast, threat or hindrance appraisals are associated with avoidance-oriented job crafting through factors including job insecurity and obsessive work passion. Importantly, these pathways are contingent upon individual-level factors (e.g., AI knowledge, positive stress mindset) and organizational-level factors (e.g., servant leadership, corporate social responsibility).

**Conclusion:**

The relationship between heterogeneous AI-related perceptions and cognitions, often broadly conceptualized as AI awareness, and job crafting appears to be contingent upon employees’ cognitive appraisals of AI. To support more adaptive and approach-oriented forms of job crafting in AI-enabled workplaces, organizations may benefit from cultivating work environments that are conducive to challenge appraisals, for example through supportive leadership, opportunities for knowledge development, and meaningful work design.

## 1 Introduction

The rapid integration of artificial intelligence (AI) into organizational life is transforming work design, reconfiguring job roles, and reshaping patterns of human–machine interaction (Daugherty and Euchner, 2020). Across sectors such as healthcare, finance, logistics, and customer service, organizations are increasingly deploying AI assistants, predictive analytics, recommendation systems, and generative AI to support a broad range of work processes (Ooi et al., 2023). This transformation has intensified markedly with the diffusion of generative AI technologies, making human–AI collaboration an increasingly constitutive feature of contemporary work systems (Caporusso, 2023). At the same time, the pace of AI adoption often exceeds organizational readiness, as technological implementation may proceed more rapidly than systematic attention to the psychological and behavioral adjustments required of employees (Landers and Marin, 2020).

As AI becomes progressively embedded in everyday work practices, understanding how employees proactively adapt to AI-enabled changes has become an increasingly important scholarly concern (Braganza et al., 2020). Existing reviews of workplace AI have predominantly emphasized organizational-level outcomes, including productivity, performance, and general attitudes toward AI, while offering comparatively limited insight into the micro-level processes through which employees interpret and respond to AI-related changes in their immediate work environments (Trenerry et al., 2021). Because AI is increasingly understood as augmenting, rather than merely replacing, human labor, effective implementation depends to a considerable extent on how employees reinterpret role expectations, adjust task boundaries, and incorporate AI into routine work practices (Tschang and Almirall, 2021). These adaptation processes make job crafting a particularly important construct for understanding employee responses to AI-enabled work transformation.

Job crafting refers to the proactive process through which employees modify the task, relational, and cognitive boundaries of their work in order to achieve a more optimal alignment between job demands, personal resources, and perceived work meaning (Wrzesniewski and Dutton, 2001; Tims et al., 2011). In AI-enabled workplaces, employees may engage in job crafting to preserve autonomy, maintain competence, manage uncertainty, or capitalize on emerging opportunities associated with technological change (Morandini et al., 2023). In this sense, job crafting is not merely a behavioral adjustment to altered work conditions, but a central mechanism through which employee agency is enacted under conditions of technological transformation. It offers a conceptually precise way to examine how employees actively reshape their work in response to AI-related demands, opportunities, and perceived disruptions.

At the same time, the emerging literature does not examine a single, uniformly defined AI-related construct. Rather, studies have operationalized what is often broadly labeled as AI awareness in heterogeneous ways, including AI exposure, perceived AI threat or opportunity, and AI-related knowledge or competence. Accumulating evidence suggests that these related but non-equivalent AI-related perceptions and cognitions may be associated with job crafting through employees’ cognitive appraisals of AI, particularly insofar as AI is interpreted as either an opportunity or a threat (Perez et al., 2022). However, these appraisal-based pathways have not yet been systematically synthesized, and the conceptual heterogeneity of AI-related constructs has not been sufficiently clarified. The present review therefore examines how heterogeneous AI-related perceptions and cognitions, often broadly grouped under the label of AI awareness, are associated with employee job crafting through cognitive appraisal processes.

## 2 Methods

This study employed a systematic review methodology following the Preferred Reporting Items for Systematic Reviews and Meta-Analyses (PRISMA 2020) guidelines (Page et al., 2021). The purpose of this review was to synthesize empirical evidence on the relationship between AI-related perceptions and cognitions, often broadly conceptualized as AI awareness in the literature, and employee job crafting, with particular emphasis on the psychological mechanisms underlying this relationship. Specifically, the review examines the mediating role of cognitive appraisal processes and the moderating influence of individual and organizational contextual factors.

AI awareness was not operationalized as a uniform construct across the reviewed studies. Rather, the literature examined a heterogeneous set of AI-related constructs, including AI exposure, perceived AI threat or opportunity, and AI-related knowledge or competence. In the present review, therefore, the term AI awareness is used as an umbrella label referring to conceptually related but non-equivalent AI-related perceptions and cognitions, rather than as a single standardized construct.

### 2.1 Literature search

A systematic literature search was conducted across four major academic databases, namely Web of Science, Scopus, PubMed, and PsycINFO. These databases were selected because they provide comprehensive coverage of high-quality peer-reviewed research across multiple disciplines relevant to the present study, including organizational psychology, management, and technology-related workplace research.

The search was restricted to peer-reviewed journal articles published in English between 2022 and 2025. This timeframe was selected because the recent emergence and rapid diffusion of generative artificial intelligence technologies have significantly accelerated the integration of AI into organizational work systems. In particular, the release of large-scale generative AI models such as ChatGPT since 2022 represents a major technological milestone that has intensified the application of AI in professional and organizational contexts (Mainardi, 2024).

Earlier research on workplace AI primarily focused on automation, algorithmic management, or broader digital transformation processes. More recent studies, however, increasingly emphasize the transformative role of AI in reshaping work processes, decision-making practices, and forms of human–technology collaboration within organizations (Machucho and Ortiz, 2025).

At the same time, the growing integration of AI technologies into everyday work practices has prompted increased scholarly attention toward understanding how employees cognitively interpret and behaviorally respond to AI-driven organizational change. Recent empirical research has begun to examine employees’ adaptive responses to AI-enabled work environments, highlighting mechanisms such as job crafting and other forms of proactive behavioral adjustment (Sha and Chai, 2025). Limiting the search to the 2022–2025 period therefore allows the present review to capture the most recent empirical evidence on employee perceptions and behavioral adaptations in the context of contemporary AI-driven workplace transformation.

The search strategy combined three groups of keywords representing the core constructs of the review and was implemented using Boolean operators. The first group included AI-related terms (e.g., artificial intelligence, AI, machine learning, generative AI, digital transformation, and algorithmic management). The second group captured employee proactive job redesign behaviors (e.g., job crafting, work redesign, task crafting, cognitive crafting, role adjustment, and proactive behavior). The third group included terms representing potential psychological mechanisms (e.g., cognitive appraisal, challenge appraisal, threat appraisal, perceived opportunity, perceived threat, job insecurity, and work engagement). Searches were conducted primarily within titles, abstracts, and keywords to ensure conceptual relevance.

### 2.2 Eligibility criteria and review focus

Studies were considered eligible if they were empirical studies involving working populations, such as employees, managers, or knowledge workers, and examined AI-related perceptions, cognitions, or experiences in workplace settings. In line with the review’s analytical focus, eligible studies were required to include job crafting or closely related crafting-oriented or adaptive work outcomes and to address cognitive appraisal variables or other relevant psychological mechanisms underlying these relationships. Quantitative, qualitative, and mixed-methods studies published in English were eligible for inclusion.

Studies were excluded if they were non-empirical publications (e.g., theoretical papers, editorials, or commentaries), focused on non-work contexts, did not involve employee-level samples, lacked relevant measures of AI-related constructs or job crafting-related outcomes, or examined only direct associations without considering appraisal-based or other mechanism-related pathways. Although this criterion was applied to maintain conceptual consistency with the mechanism-focused aim of the review, it may have reduced the coverage of studies reporting only direct associations and, consequently, limited the extent to which mechanism-based and non-mechanism-based evidence could be compared. Duplicate publications and conference abstracts without full-text availability were also excluded.

### 2.3 Study selection process

**Figure 1:**
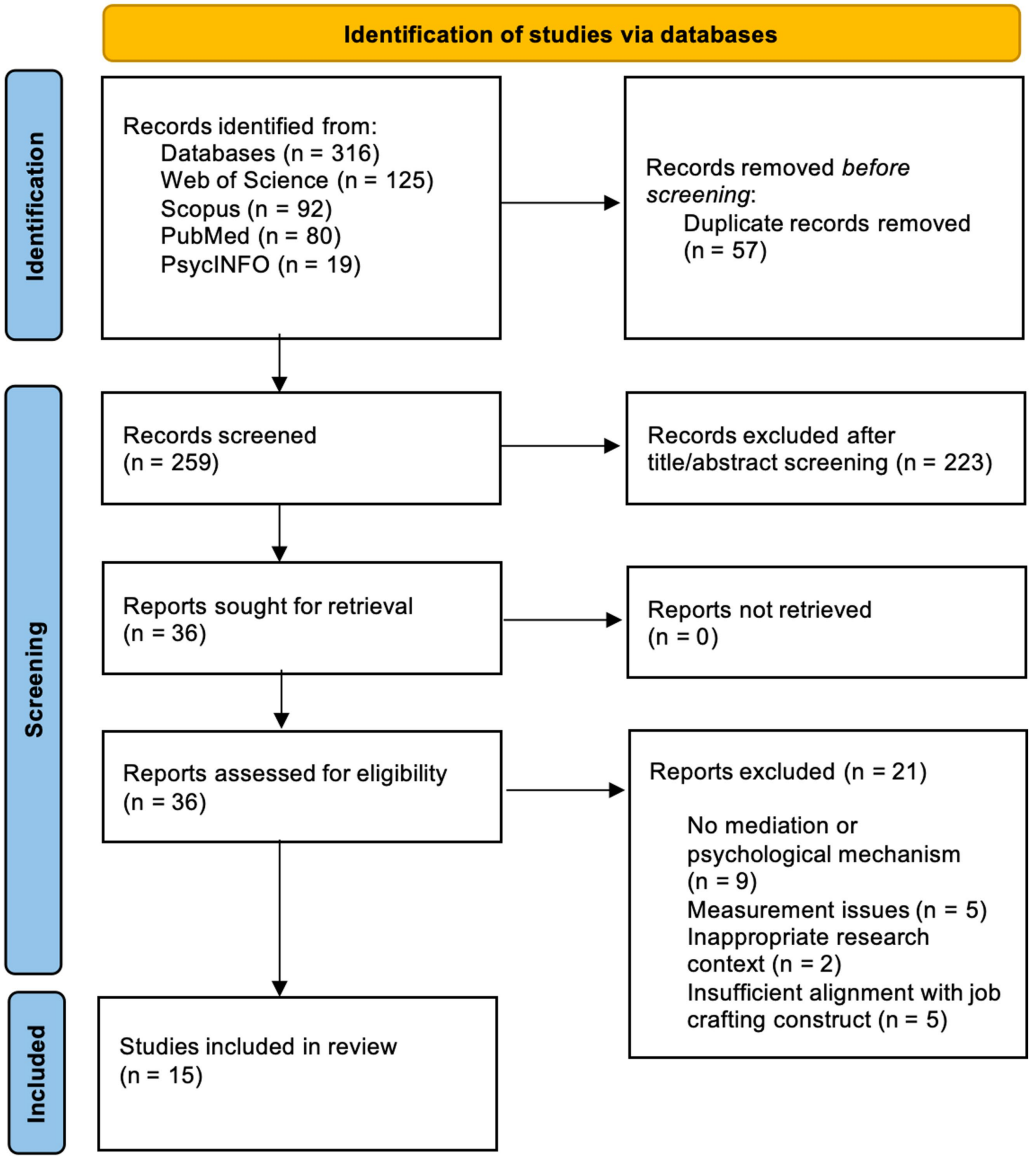
PRISMA 2020 flow diagram of the literature selection process.

The study selection process followed the PRISMA 2020 guidelines. A total of 316 records were identified through database searching across Web of Science (*n* = 125), Scopus (*n* = 92), PubMed (*n* = 80), and PsycINFO (*n* = 19). After removal of 57 duplicates, 259 records remained for title and abstract screening. During this stage, 223 records were excluded because they did not meet the predefined eligibility criteria, primarily due to lack of relevance to workplace AI contexts, non-empirical study designs, non-working populations, or the absence of job crafting-related outcomes. The remaining 36 articles were retrieved for full-text assessment. Following full-text review, 21 studies were further excluded because they did not address appraisal-based or other relevant psychological mechanisms, showed insufficient conceptual alignment with the review focus, or presented substantial construct-operationalization issues. Ultimately, 15 studies met all eligibility criteria and were included in the final systematic review and narrative synthesis. Figure 1 presents the PRISMA 2020 flow diagram of the study selection process.

### 2.4 Data extraction and synthesis

Title and abstract screening, full-text assessment, data extraction, and synthesis were conducted primarily by the first author in accordance with the predefined eligibility criteria and a structured coding framework. To strengthen the methodological transparency and consistency of the screening process, an inter-rater reliability check was conducted at the title and abstract screening stage. After duplicate removal, 259 records remained for initial screening. From this pool, a random subsample of 60 records was selected and independently assessed by a second reviewer using the same predefined eligibility criteria as the first reviewer. At this stage, records were retained when the title or abstract indicated that the study concerned AI, algorithmic systems, or human–AI interaction in workplace or organizational contexts, involved employee or work-related samples, and examined job crafting or closely related proactive or adaptive work outcomes. Records were excluded when they were clearly theoretical or review-based rather than empirical, did not involve workplace settings or employee samples, or addressed only general attitudes, performance, or well-being without reference to job crafting or related adaptive work adjustment. Inter-rater reliability was quantified in R using Cohen’s kappa, with percentage agreement reported as a complementary index. The agreement between the two reviewers was substantial (*κ* = 0.71, *p* < 0.001), and the percentage agreement was 90.0%, indicating a satisfactory level of consistency in the application of the screening criteria. Further details of the screening decision distribution are provided in Table 1.

**Table 1:** Inter-rater reliability for title and abstract screening based on a random subsample of records (*n* = 60).

Reviewer 1 decision	Reviewer 2 decision	Number of records
Include	Include	19
Include	Exclude	3
Exclude	Include	3
Exclude	Exclude	35
Total		60

Percentage agreement: 90%.

Cohen’s κ: 0.71 (*p* < 0.001).

Extracted data included study characteristics, sample and contextual information, AI-related constructs, appraisal variables, job crafting dimensions and related adaptive outcomes, measurement instruments, analytical approaches, and principal findings. Owing to substantial heterogeneity in construct definitions, measurement strategies, and analytical approaches across the included studies, the findings were synthesized using a structured narrative approach supplemented by subgroup-based comparison rather than formal quantitative synthesis. Extracted information was cross-checked to enhance accuracy and completeness. Regarding outcome coding and conceptual mapping, synthesized outcomes were further classified to address heterogeneity in operationalization across studies. We distinguished between direct measures of job crafting and crafting-adjacent adaptive behaviors. Direct job crafting outcomes included task, relational, or cognitive modifications explicitly framed as employee-initiated changes to work roles or boundaries. Crafting-adjacent outcomes encompassed proactive behaviors, learning engagement, withdrawal, avoidance, or role-protective strategies that reflected employees’ active regulation of task demands and resources in response to AI-related changes. Consistent with approach–avoidance models of self-regulation, these outcomes were coded as approach-oriented or avoidance-oriented forms of adaptive work regulation, enabling integrative synthesis across studies. The synthesized results are presented in Table 2.

**Table 2:** Description of job crafting scales and statistical methods used across included studies.

Study	Type of study	Job crafting scale used	Job crafting measured	Statistical method
Xu et al. (2025)	Quantitative (survey)	16-item promotion-focused and 12-item prevention-focused job crafting scale (Bindl et al., 2019)	Promotion- and prevention-focused job crafting	Path analysis; bootstrapped mediation (Mplus 8.3)
Yan et al. (2025)	Quantitative (survey)	(Tims et al., 2011) Job Crafting Scale was used, which consists of two subscales: approach crafting with 15 items, and avoidance crafting with 6 items	Approach job crafting; avoidance job crafting	Hierarchical regression; mediated moderation; CFA (Mplus 8.3)
Dong et al. (2024)	Quantitative (survey)	A six-item AI crafting scale adapted from the original job crafting framework to assess employees’ proactive adjustments to AI in work settings (Leana et al., 2009; Li et al., 2024)	AI crafting (proactive adjustments in AI-enabled work)	Regression-based mediation/moderated mediation; CFA
Sha and Chai (2025)	Quantitative (two-wave survey)	Four-item scale developed by Leana et al. (2009)	Approach-oriented job crafting	SEM; moderated mediation (PROCESS)
Qin et al. (2025)	Scenario-based experiment	No standardized job crafting scale; behavioral response indicators	Adaptive: exploitative/exploratory learning; maladaptive: knowledge/information withholding	PLS-SEM
Li et al. (2025)	Quantitative two-wave field survey	Proactive behavior was measured using an established nine-item scale (Griffin et al., 2007). Withdrawal behavior was assessed with a validated twelve-item instrument (Lehman and Simpson, 1992).	Approach-oriented crafting as Proactive Behavior. Avoidance-oriented crafting as Withdrawal Behavior	Moderated mediation (PROCESS; bootstrapping); CFA; correlations
Einola and Khoreva (2022)	Qualitative (case study)	No standardized scale; job crafting derived from interview/field data	Approach- and avoidance-oriented job crafting themes	Thematic analysis; interpretive/hermeneutic analysis; triangulation/cross-checking
Emilisa et al. (2024)	Quantitative cross-sectional survey study	The Job Crafting Scale developed by Emilisa et al. (2024) is a 13-item instrument grounded in a multidimensional framework	The scale measures proactive job redesign through three dimensions: Task Crafting, Relationship Crafting, and Cognitive Crafting	SEM with path analysis using AMOS, supplemented by the Sobel test for mediation analysis
Adya and Desiana (2025)	Quantitative (cross-sectional survey)	Job crafting was measured using a validated four-item scale adapted from established multidimensional frameworks, capturing both approach-oriented and avoidance-oriented forms of crafting	Job crafting as mediator (task/relational/cognitive adjustments)	SEM (LISREL 8.51); product-of-coefficients mediation
Lestari et al. (2024)	Quantitative (cross-sectional survey)	A 17-item, researcher-developed scale covering task, relational, and cognitive crafting behaviors	Job crafting (treated as holistic mediator)	SEM; Sobel test (mediation)
Perez et al. (2022)	Qualitative (interviews)	Not applicable (qualitative study based on Wrzesniewski and Dutton, 2001)	Task/relational/cognitive job crafting in response to AI changes	Semi-structured interviews; inductive thematic analysis; coding of crafting forms
Cheng et al. (2023)	Quantitative empirical study (three-wave time-lagged design)	16-item promotion-focused and 12-item prevention-focused job crafting scale (Bindl et al., 2019)	Supervisor-rated promotion-focused and prevention-focused job crafting (task, relational, skill, and cognitive crafting via parceling)	Latent moderated structural equation modeling (LMS) with bootstrapped moderated mediation analysis in Mplus
He et al. (2024)	Quantitative (cross-sectional survey)	4-item job crafting scale (Leana et al., 2009)	Job crafting (proactive adjustment of work processes/methods)	Structural equation modeling with mediation and moderation analyses (challenge–hindrance appraisal model)
Wang and Li (2025)	Quantitative (survey)	Job Crafting Scale (Tims et al., 2011); employee-rated	Approach-oriented job crafting (task redesign/skill development/role adaptation)	SEM; mediation (coping mechanisms)
Perez et al. (2024)	Qualitative (multi-method case study)	Not applicable (qualitative study grounded in Wrzesniewski and Dutton, 2001)	Approach/avoidance job crafting (task/relational/identity crafting)	Semi-structured interviews, observations, and document analysis; inductive thematic analysis with systematic coding of job crafting forms

### 2.5 Sensitivity analysis

Given the small number of included studies and substantial heterogeneity in construct operationalization, study design, analytical approach, and outcome definition, formal quantitative sensitivity analyses were not feasible. The studies did not provide sufficiently comparable effect size estimates, and both AI-related constructs and job crafting were operationalized inconsistently across the evidence base. Accordingly, a subgroup-based narrative sensitivity analysis was conducted to examine whether the overall synthesis remained broadly stable across key conceptual and methodological variations.

Studies were compared across four dimensions: type of AI-related construct, operationalization of job crafting and related adaptive responses, study design and methodological profile, and outcome definition. These subgroup comparisons are summarized in Table 3.

**Table 3:** Narrative sensitivity comparison across key conceptual and methodological subgroups.

Subgroup dimension	Categories compared	Summary of subgroup comparison	Implication for the main synthesis
Type of AI-related construct	Exposure-based indicators, evaluative perceptions, and capability-related indicators	Across all three categories, direct associations with job crafting were generally weak, inconsistent, or attenuated once appraisal-related variables were considered. Evaluative perceptions appeared somewhat more proximally related to differentiated adaptive responses.	The central appraisal-linked interpretation remained broadly stable.
Job crafting operationalization	Global measures, dimension-specific measures, and crafting-adjacent adaptive outcomes	The overall pattern was substantively similar across operationalizations, although studies using differentiated dimensions revealed greater nuance in approach-oriented and avoidance-oriented responses.	No material change in the main synthesis was observed.
Study design and methodological profile	Cross-sectional quantitative studies and qualitative or process-oriented studies	Cross-sectional studies most often reported mediated associative patterns, whereas qualitative studies provided convergent interpretive evidence regarding employee sense-making and adaptive adjustment.	The overall narrative remained convergent, although causal inference remained limited.
Outcome definition	Explicit job crafting outcomes and broader adaptive-behavior outcomes	Greater emphasis on studies using explicit job crafting measures did not materially alter the overall interpretation.	No material change in the main synthesis was observed.

This table summarizes a subgroup-based narrative sensitivity analysis rather than a formal quantitative sensitivity analysis. The comparisons were conducted to assess whether the principal interpretive pattern of the review remained substantively stable across major sources of conceptual and methodological heterogeneity. Given the limited number of included studies and the lack of sufficiently comparable effect size estimates, these comparisons should be interpreted as exploratory.

This procedure was used to assess whether the principal interpretive pattern identified in the review—namely, that heterogeneous AI-related perceptions and cognitions were more consistently associated with employee adaptation through appraisal-related pathways than through stable direct associations—remained substantively similar across subgroups. Given the small number of studies within each subgroup, the analysis was interpreted as exploratory rather than confirmatory.

### 2.6 Risk of Bias assessment

The methodological quality and risk of bias of the included studies were assessed using the Mixed Methods Appraisal Tool (Hong et al., 2018), which is suitable for systematic reviews encompassing qualitative, quantitative, and mixed-methods research. Given the heterogeneity of study designs and the predominance of observational and qualitative approaches in the included literature, risk of bias was evaluated according to study type using the corresponding MMAT criteria.

For quantitative studies, the appraisal focused on sampling strategies, participant representativeness, validity of measurement instruments, consideration of potential confounding variables, and the appropriateness of statistical analyses. Qualitative studies were evaluated based on the adequacy of data collection methods, coherence between data and interpretations, and transparency of analytic procedures. Mixed-methods studies were appraised with particular attention to the rationale for methodological integration and the consistency between qualitative and quantitative components.

Each MMAT criterion was rated as “Yes,” “No,” or “Cannot tell,” and an overall judgment of low, unclear, or high risk of bias was assigned to each study. The results of the risk of bias assessment are summarized in Table 4. Risk of bias assessment was conducted alongside data extraction, and any discrepancies were resolved through discussion and consensus.

**Table 4:** Risk of bias assessment of included studies using MMAT (2018).

Panel A. Quantitative studies						
Study	Sampling representativeness	Measures appropriate	Outcome data complete	Confounding control	Analysis appropriate	Overall risk
Xu et al. (2025)	CT	Y	Y	CT	Y	Unclear
Yan et al. (2025)	CT	Y	Y	CT	Y	Unclear
Dong et al. (2024)	CT	Y	Y	CT	Y	Unclear
Sha and Chai (2025)	CT	Y	Y	CT	Y	Unclear
Qin et al. (2025)	CT	CT	CT	CT	Y	Unclear
Li et al. (2025)	CT	Y	Y	CT	Y	Unclear
Emilisa et al. (2024)	CT	Y	Y	CT	Y	Unclear
Adya and Desiana (2025)	CT	CT	CT	CT	Y	Unclear
Lestari et al. (2024)	N	CT	CT	CT	Y	High
Cheng et al. (2023)	CT	Y	Y	CT	Y	Unclear
He et al. (2024)	CT	Y	Y	CT	Y	Unclear
Wang and Li (2025)	CT	Y	Y	CT	Y	Unclear

Panel B. Qualitative studies						
Study	Qualitative approach appropriate	Data collection adequate	Findings grounded in data	Interpretation substantiated	Coherence	Overall risk
Einola and Khoreva (2022)	Y	CT	Y	CT	Y	Unclear–Low
Perez et al. (2022)	Y	Y	Y	Y	Y	Low
Perez et al. (2024)	Y	Y	Y	CT	Y	Unclear–Low

Risk of bias was assessed using the Mixed Methods Appraisal Tool (MMAT; Hong et al., 2018). Criteria were applied according to study type. Y, Yes; N, No; CT, Cannot tell. Overall risk judgments reflect the completeness and transparency of methodological reporting.

A formal GRADE assessment was not undertaken because the evidence base was limited in size and characterized by substantial heterogeneity in study design, construct operationalization, analytical approach, and outcome definition (Guyatt et al., 2008). In addition, the included studies did not provide sufficiently comparable effect size estimates to support a conventional certainty-of-evidence rating. Under these conditions, evidence strength and interpretive stability were evaluated through MMAT-based quality appraisal, subgroup-based narrative sensitivity analysis, and cautious interpretation of study design limitations.

## 3 Results

### 3.1 Risk of bias and methodological quality of included studies

Risk of bias and methodological quality were assessed using the Mixed Methods Appraisal Tool (Hong et al., 2018). Given the inclusion of both quantitative and qualitative studies, individual MMAT criteria were not reported verbatim but were conceptually synthesized and mapped onto five overarching methodological domains to facilitate cross-study comparison. These domains captured key aspects of study quality, including sampling or case selection adequacy, measurement and data collection appropriateness, outcome data completeness or saturation, analytical rigor and confounding control, and reporting transparency or coherence.

**Figure 2:**
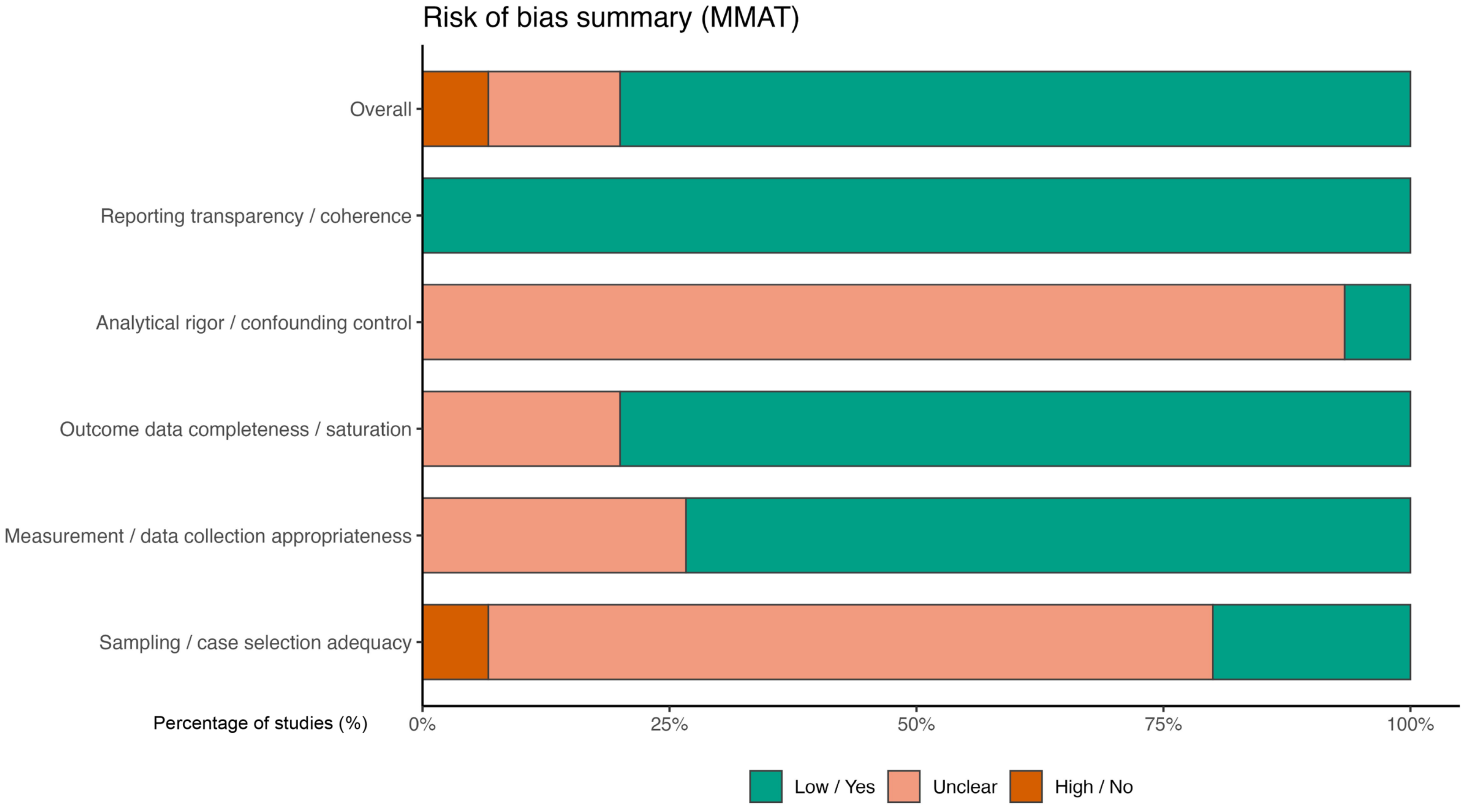
Summary of risk-of-bias judgments across domains.

Figure 2 summarizes the overall distribution of risk-of-bias judgments across these domains. Across the included studies, judgments indicating low methodological risk predominated in domains related to measurement quality and reporting transparency. In contrast, a higher proportion of unclear judgments was observed for domains pertaining to sampling strategies and analytical rigor, reflecting limited reporting detail or insufficient information regarding case selection procedures and control of confounding factors. Explicit high-risk judgments were relatively rare and confined to a small number of studies.

**Figure 3:**
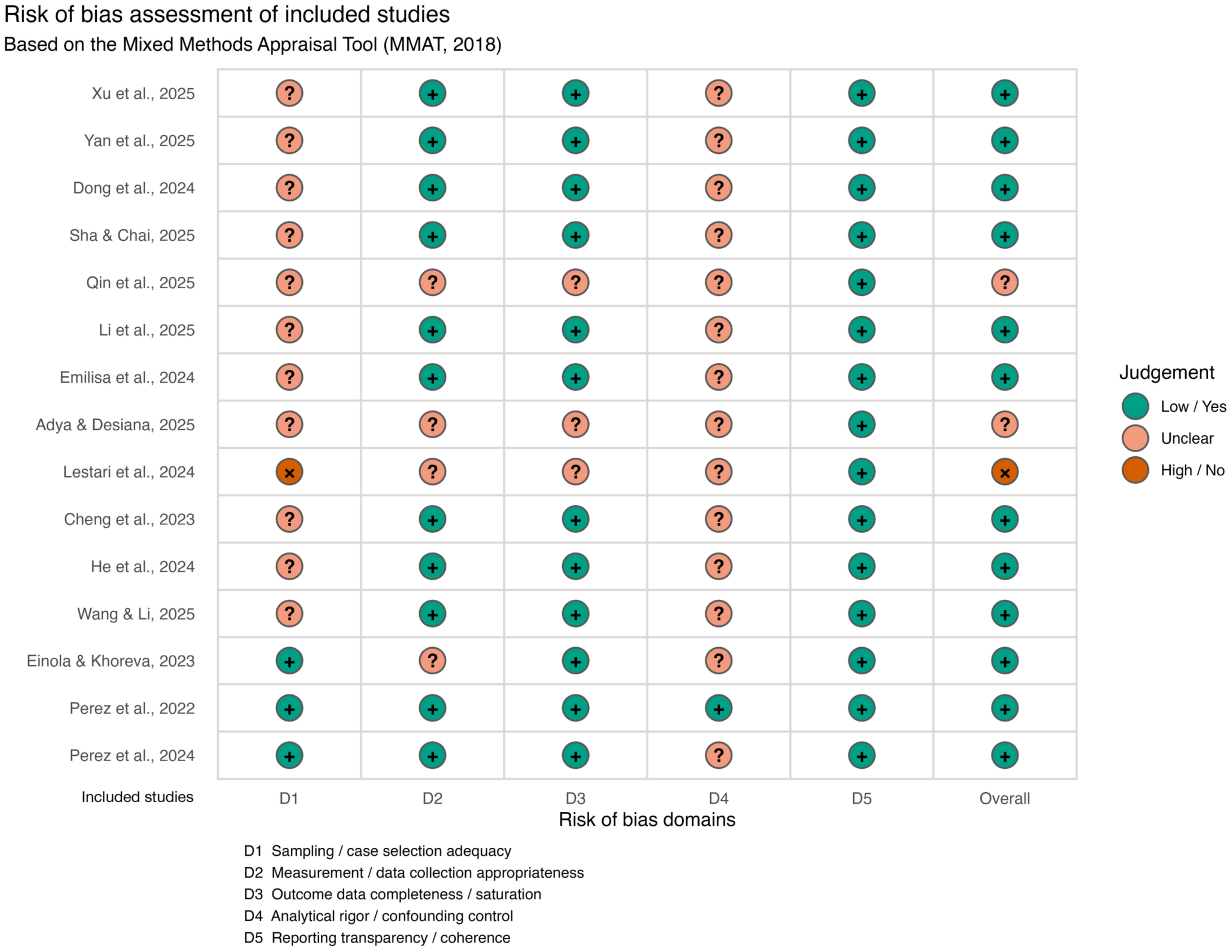
Study-level risk-of-bias assessment (traffic-light plot).

Study-level assessments are presented in Figure 3 using a traffic-light visualization. This figure illustrates considerable heterogeneity in methodological profiles across individual studies, with several studies exhibiting mixed patterns of low and unclear risk across domains. Importantly, unclear judgments predominantly reflected ambiguity or incomplete reporting rather than clearly identifiable methodological flaws.

The overall assessment suggests a generally moderate to good level of methodological quality across the included studies, with variability arising mainly from incomplete reporting and design-related limitations rather than systematic sources of bias. Accordingly, findings related to appraisal–job crafting pathways should be interpreted cautiously in domains exhibiting higher uncertainty.

### 3.2 Appraisal-based associations between heterogeneous AI-related perceptions and cognitions and job crafting

Across the reviewed studies, the focal AI-related constructs varied in their operationalization. For interpretive clarity, these constructs can be broadly grouped into three categories: exposure-based indicators (e.g., AI exposure), evaluative perceptions (e.g., perceived AI threat or opportunity), and capability-related indicators (e.g., AI-related knowledge or competence). A study-level mapping of these operational differences is presented in Table 5, which summarizes how AI-related constructs and job crafting and related outcomes were operationalized across the included studies.

**Table 5:** Operational heterogeneity of AI-related constructs and job crafting-related outcomes across included studies.

Study	AI-related construct	Category	AI operationalization	Job crafting and related outcomes	Core finding
Xu et al. (2025)	AI challenge appraisal, AI hindrance appraisal, and AI knowledge	Evaluative appraisal + capability-related resource	4-item AI challenge appraisal scale, 3-item AI hindrance appraisal scale, and 5-item AI knowledge scale	Promotion-focused and prevention-focused job crafting	AI challenge appraisal was positively associated with promotion-focused job crafting, whereas AI hindrance appraisal was positively associated with prevention-focused job crafting; AI knowledge strengthened the challenge-appraisal pathway.
Yan et al. (2025)	GenAI awareness	Evaluative perception	4-item GenAI awareness scale; internal CSR, external CSR, harmonious passion, and obsessive passion in a three-wave moderated mediation design	Approach job crafting and avoidance job crafting	GenAI awareness showed an indirect association with approach job crafting via harmonious passion under high internal CSR and with avoidance job crafting via obsessive passion under high external CSR.
Dong et al. (2024)	AI talk, leader AI-focused attention, and AI self-efficacy	Exposure−/communication-based + capability-related mediator	5-item AI talk scale, 5-item leader AI-focused attention scale, and 3-item AI self-efficacy scale	AI crafting	AI talk was positively associated with AI crafting through AI self-efficacy; leader AI-focused attention strengthened the AI talk–AI self-efficacy association.
Sha and Chai (2025)	Digital-AI transformation and AI knowledge	Exposure−/transformation-based + capability-related resource	3-item digital-AI transformation scale and 5-item AI knowledge scale	General job crafting	Digital-AI transformation was positively associated with job crafting, and AI knowledge strengthened this adaptive pathway.
Qin et al. (2025)	AI awareness as challenge versus hindrance stressor	Evaluative appraisal	Stress-appraisal framing of AI awareness in a human-AI symbiosis context	Job crafting-related adaptive and maladaptive behavioral responses	Challenge-oriented AI awareness was associated with more adaptive responses, whereas hindrance-oriented AI awareness was associated with less adaptive responses.
Li et al. (2025)	AI awareness as opportunity- and threat-related perception	Evaluative perception	AI awareness scale derived from Brougham and Haar, 2017; opportunity- and threat-related interpretations linked to proactive and withdrawal responses	Job crafting-related approach and avoidance behavioral responses	Opportunity-oriented AI awareness was associated with more proactive responses, whereas threat-oriented AI awareness was associated with more withdrawal-oriented responses.
Einola and Khoreva (2022)	Employee sensemaking of AI	Interpretive qualitative	Qualitative analysis of employee interpretations of AI as both enabling and constraining	Qualitatively identified adaptive adjustment responses	Employees described AI as simultaneously enabling and constraining and reported mixed adaptive responses to AI-related work change.
Emilisa et al. (2024)	AI challenge appraisal and AI hindrance appraisal	Evaluative appraisal	4-item AI challenge appraisal scale and 3-item AI hindrance appraisal scale	Multidimensional job crafting	AI challenge appraisal was positively associated with job crafting, whereas AI hindrance appraisal was negatively associated with job crafting.
Adya and Desiana (2025)	STARA challenge appraisal and STARA hindrance appraisal	Evaluative appraisal	STARA awareness operationalized as challenge versus hindrance appraisal toward STARA	General job crafting	Challenge appraisal toward STARA showed a positive indirect association with service performance through job crafting and work engagement, whereas hindrance appraisal was linked to the negative pathway.
Lestari et al. (2024)	AI awareness and servant leadership	Evaluative perception + contextual factor	Survey-based model including AI awareness and servant leadership as antecedents of job crafting and work engagement	General job crafting	Servant leadership was positively associated with job crafting, whereas the association between AI awareness and job crafting was weaker.
Perez et al. (2022)	AI-driven changes to work	Qualitative technology-change	Qualitative interviews examining how learning algorithms altered work experience, autonomy, and meaning	Task, relational, and cognitive crafting	Employees reported task, relational, and cognitive crafting in response to AI-related changes in work design.
Cheng et al. (2023)	Organizational AI adoption	Exposure−/context-based	3-item organizational AI adoption scale; challenge appraisal and hindrance appraisal as mediators; internal and external locus of control as moderators	Promotion-focused and prevention-focused job crafting	Organizational AI adoption showed an indirect association with promotion-focused job crafting via challenge appraisal and with prevention-focused job crafting via hindrance appraisal, conditional on locus of control.
He et al. (2024)	AI challenge appraisal, AI hindrance appraisal, and AI knowledge	Evaluative appraisal + capability-related resource	Survey-based measures of AI challenge appraisal, AI hindrance appraisal, and AI knowledge	General job crafting	AI challenge appraisal was positively associated with job crafting, whereas AI hindrance appraisal did not show a comparable positive association with job crafting.
Wang and Li (2025)	AI challenge perception and AI threat perception	Evaluative perception	AI stress perception adapted from Brougham and Haar, 2017; challenge and threat dimensions examined alongside coping variables	Approach-oriented job crafting	Challenge-oriented AI perception was positively associated with problem-focused coping and approach-oriented job crafting, whereas threat-oriented AI perception was more closely aligned with emotion-focused coping.
Perez et al. (2024)	AI-related identity threat and interpretation	Interpretive qualitative	Multi-level qualitative interviews examining perceptions of AI and responses to AI-related professional change	Approach crafting, avoidance crafting, and identity crafting	Participants reported approach crafting, avoidance crafting, and identity crafting in response to AI-related work change.

Outcome operationalizations included both direct job crafting measures and conceptually related adaptive or maladaptive work responses used in the reviewed mechanism-focused studies.

Across the included quantitative studies, these heterogeneous AI-related perceptions and cognitions did not demonstrate consistent direct associations with job crafting once cognitive appraisal variables were incorporated into the analytical models. Several studies reported that the direct associations between these AI-related constructs and job crafting became weak or non-significant when appraisal-based mediators were included (e.g., Cheng et al., 2023; Li et al., 2025; He et al., 2024), suggesting that these constructs may function more as distal contextual correlates of employees’ appraisals than as variables showing stable direct associations with job crafting.

A convergent pattern emerged in which cognitive appraisals were repeatedly examined as mediating variables in the relationship between AI awareness and differentiated job crafting responses. When AI was appraised as a challenge—such as an opportunity for learning, performance enhancement, or professional development—employees were more likely to engage in approach-oriented job crafting. This indirect pathway was supported across studies employing diverse operationalizations of job crafting, including promotion-focused crafting (Cheng et al., 2023; Xu et al., 2025), proactive task redesign (He et al., 2024), and multidimensional job crafting behaviors (Emilisa et al., 2024). Mediation analyses further suggested that challenge appraisals may function as intermediary variables linking AI awareness with job crafting, with work engagement, creative process engagement, and harmonious work passion frequently examined as related explanatory processes (Xu et al., 2025; Emilisa et al., 2024).

In contrast, threat or hindrance appraisals of AI were consistently associated with avoidance-oriented job crafting and withdrawal-related outcomes. Studies incorporating job insecurity as a mediating variable suggest that higher levels of AI-related threat salience may be associated with stronger concerns about employment stability, which are, in turn, linked to lower levels of proactive adaptation and greater endorsement of withdrawal-related or defensive coping responses (Li et al., 2025; Adya and Desiana, 2025). Similarly, Cheng et al. (2023) reported that prevention-focused job crafting was more likely when AI-related changes were perceived as threatening rather than challenging. Experimental evidence further supported this pattern, indicating that perceived AI-related threats were associated with maladaptive behavioral responses, including knowledge withholding and reduced exploratory learning (Qin et al., 2025).

Taken together, evidence across the quantitative studies is broadly consistent with a dual appraisal-based pattern in which AI awareness is associated with approach-oriented job crafting through challenge appraisals and with avoidance-oriented responses through threat appraisals.

### 3.3 Boundary conditions of appraisal–job crafting relationships

Beyond the core appraisal pathways, several studies identified individual- and organizational-level moderators that shaped the strength of appraisal–job crafting relationships. These boundary conditions suggest that the association between cognitive appraisals and job crafting may vary depending on personal resources and contextual support.

At the individual level, AI-related knowledge and learning orientation consistently emerged as buffering resources. Employees with higher levels of AI knowledge were more likely to report approach-oriented job crafting in the context of challenge appraisals, and less likely to report negative behavioral correlates of threat appraisals (He et al., 2024; Cheng et al., 2023). Similarly, a positive stress mindset and adaptive coping orientations appeared to be associated with stronger indirect pathways linking challenge appraisals to proactive job crafting (Xu et al., 2025). In contrast, lower levels of perceived competence in dealing with AI intensified threat appraisals and reinforced avoidance-oriented responses (Adya and Desiana, 2025).

Organizational context further moderated appraisal-based pathways. Supportive leadership styles and favorable organizational climates were associated with weaker threat appraisals and greater proactive adaptation. For example, servant leadership was found to attenuate the relationship between AI-related threat appraisals and avoidance behaviors and was also linked to higher levels of job crafting in models incorporating work engagement (Lestari et al., 2024). Studies examining organizational signals such as corporate social responsibility and ethical AI practices similarly indicated that supportive contexts were associated with stronger challenge appraisals and reduced threat-based responses (Emilisa et al., 2024; Dong et al., 2024).

These findings indicate that appraisal–job crafting relationships are not uniform but are shaped by the availability of individual and organizational resources that influence how AI-related demands are interpreted and addressed.

### 3.4 Qualitative evidence and integrated empirical patterns

Qualitative studies provided complementary evidence on the appraisal-driven nature of AI-induced job crafting by illuminating how employees make sense of AI in everyday work contexts. Employees were often found to perceive AI simultaneously as both enabling and constraining, which coincided with the coexistence of approach-oriented and avoidance-oriented job crafting within the same organizational context (Einola and Khoreva, 2022). These findings illustrate how ambivalent appraisals can be associated with dynamic and sometimes contradictory crafting strategies over time.

Perez et al. reported that employees responded to AI-driven workplace transformation through task, relational, and cognitive job crafting, with these adaptive responses shaped by evolving interpretations of AI’s implications for work. Their subsequent multi-method case study further suggested that identity crafting constituted an additional form of adaptation, illustrating how employees reconstructed their sense of professional self to maintain meaning and legitimacy in algorithmically mediated work settings (Perez et al., 2022; Perez et al., 2024).

Synthesizing quantitative and qualitative evidence reveals a coherent empirical pattern whereby heterogeneous AI-related perceptions and cognitions are associated with cognitive appraisal processes that shape both approach-oriented and avoidance-oriented job crafting trajectories. Importantly, these patterns do not reflect a single uniformly operationalized AI awareness construct but rather represent a broader family of related AI-oriented perceptions, evaluations, and knowledge-based indicators. While quantitative studies provide convergent evidence for appraisal-based associations, qualitative findings further enrich this understanding by illuminating the content, dynamics, and identity-related dimensions of appraisal and crafting processes over time.

## 4 Discussion

### 4.1 Appraisal-linked patterns of job crafting in AI-related contexts

This systematic review synthesizes evidence from 15 empirical studies to examine how heterogeneous AI-related perceptions and cognitions, often broadly grouped under the label of AI awareness in the literature, are associated with employee job crafting, with particular attention to the role of cognitive appraisal processes. Across the reviewed studies, the AI-related constructs examined did not show stable or consistently robust direct associations with job crafting once appraisal-based variables were taken into account. This pattern suggests that these AI-related perceptions and cognitions may function more as distal contextual conditions than as proximal correlates of job crafting (Cheng et al., 2023; Li et al., 2025; He et al., 2024). Within the bounds of the included evidence, challenge- and threat-based interpretations of AI appeared to be recurrent psychological processes associated with differentiated job crafting responses.

The reviewed evidence further suggests that challenge appraisals of AI are often associated with approach-oriented forms of job crafting, including proactive task redesign, skill development, and role expansion (Cheng et al., 2023; Xu et al., 2025; Emilisa et al., 2024). These patterns were frequently reported alongside engagement-related variables, including work engagement, creative process engagement, and harmonious work passion (Xu et al., 2025; Emilisa et al., 2024). By contrast, threat- or hindrance-oriented appraisals, often linked to job insecurity and perceived loss of control, were more commonly associated with avoidance-oriented job crafting and withdrawal-related responses (Li et al., 2025; Adya and Desiana, 2025; Qin et al., 2025). Importantly, the avoidance-oriented outcomes identified in this review should not be interpreted solely as passive disengagement. Rather, they may also reflect protective or defensive forms of work regulation through which employees attempt to preserve control under perceived AI-related threat. Taken together, these findings extend current interpretations of job crafting in AI-related contexts by suggesting that approach-oriented and avoidance-oriented forms of adaptation may be linked to distinguishable patterns of cognitive evaluation rather than representing undifferentiated variations in proactive behavior.

The overall evidence base should nevertheless be interpreted with caution. Although MMAT-based appraisal and subgroup-based narrative sensitivity analysis indicated that the principal interpretive pattern was broadly robust across key conceptual and methodological variations, the predominance of cross-sectional designs, the limited number of included studies, and substantial heterogeneity in construct operationalization and outcome definition preclude any high-certainty interpretation (Hong et al., 2018). Furthermore, because the review was restricted to mechanism-oriented studies, the present synthesis should not be read as a comprehensive assessment of all possible direct associations between AI-related constructs and job crafting. This eligibility decision may also have reduced the coverage of studies reporting only direct associations, thereby limiting comparison between mechanism-based and non-mechanism-based evidence. The review therefore offers cautious interpretive support for appraisal-linked associative patterns rather than definitive evidence of stable causal mechanisms.

### 4.2 Boundary conditions and conceptual clarification

Several reviewed studies also suggest that appraisal–job crafting relationships may be contingent upon both individual and organizational conditions. At the individual level, resources such as AI-related knowledge and a more positive stress mindset were associated with stronger challenge-oriented patterns and weaker threat-related responses (He et al., 2024; Xu et al., 2025). At the organizational level, supportive leadership and favorable organizational conditions were linked to weaker threat appraisals and more adaptive job crafting tendencies (Lestari et al., 2024; Dong et al., 2024; Emilisa et al., 2024). Qualitative evidence complements these findings by illustrating how ambivalent appraisals of AI may give rise to dynamic combinations of approach-oriented and avoidance-oriented crafting strategies, including identity-related adjustments (Einola and Khoreva, 2022; Perez et al., 2022; Perez et al., 2024). At the same time, these patterns should be interpreted in light of the strong concentration of the current evidence base in collectivist contexts, particularly China and Indonesia, which may shape both AI-related appraisals and preferred forms of adaptive work regulation.

These findings should therefore not be interpreted as evidence concerning a single, psychometrically uniform AI awareness construct. Rather, they suggest that conceptually related but operationally distinct AI-related perceptions and cognitions may converge in their associations with appraisal processes, which are in turn linked to differentiated job crafting responses. This interpretation offers a more cautious and conceptually precise account of how employees may respond to AI-related workplace change while also underscoring the need for future research using more standardized constructs, longitudinal designs, and broader cultural samples.

### 4.3 Limitations and future research directions

Several limitations inherent in the current body of evidence should be considered when interpreting the findings of this review. The empirical literature is heavily dominated by cross-sectional, self-report research designs, which constrain the extent to which causal relationships can be inferred and raise the possibility of common method bias. Consequently, the relationships synthesized in this review should be interpreted primarily as associative patterns rather than definitive causal mechanisms. Although a small number of studies have employed time-lagged or experimental approaches, robust longitudinal evidence examining the temporal dynamics of AI-related perceptions and job crafting remains limited.

Methodological transparency within the existing literature also warrants attention. The risk-of-bias assessment indicated that a substantial proportion of the quantitative studies were rated as having an unclear risk of bias, largely due to insufficient reporting of sampling procedures and limited consideration of potential confounding variables. As a result, the magnitude of the observed associations and the inferred appraisal pathways should be interpreted with appropriate caution.

The qualitative evidence base remains comparatively small. While qualitative studies offer valuable insight into the subjective meanings, interpretive processes, and contextual dynamics underlying employee adaptation to AI-enabled work environments, the limited number of such investigations constrains the generalizability of process-oriented insights across occupational settings and cultural contexts. Expanding the use of qualitative and mixed-methods approaches would therefore provide a richer understanding of how cognitive appraisal and job crafting processes unfold and evolve over time.

An additional consideration concerns the geographical distribution of the existing empirical literature. A large proportion of the included studies were conducted in collectivist cultural contexts, particularly in China and Indonesia, whereas empirical evidence derived from Western organizational settings remains comparatively limited. This imbalance may partly reflect the rapid digital transformation and accelerating adoption of AI technologies across emerging Asian economies, which has attracted increasing scholarly attention (Sulehri et al., 2025). At the same time, cultural value orientations are likely to shape how employees cognitively interpret and behaviorally respond to AI-enabled work environments (Hofstede, 2001). In collectivist contexts, stronger norms of organizational alignment and group-oriented adjustment may encourage adaptive behavioral responses to technological change, whereas employees in more individualistic cultures may interpret AI-related transformations through different motivational or autonomy-related frameworks.

These contextual differences suggest that caution is warranted when generalizing the present findings across cultural settings. Future research would benefit from incorporating comparative cross-cultural designs to examine the boundary conditions under which AI-related cognitive appraisal processes translate into distinct job crafting strategies.

Finally, several theoretically relevant dimensions of proactive adaptation in AI-enabled workplaces remain underexplored. Emerging research has begun to highlight the importance of identity-related adaptation, moral positioning toward algorithmic systems, and collective forms of job crafting that unfold at the team or organizational level. Integrating these perspectives with longitudinal and multilevel research designs would contribute to a more comprehensive understanding of how employees navigate the psychological and behavioral challenges associated with the increasing integration of artificial intelligence into contemporary work systems.

### 4.4 Theoretical contributions

The present review advances the literature by positioning AI-related workplace change not simply as a contextual disruption, but as a condition under which employees actively reshape their work through differentiated forms of adaptation. In doing so, it situates job crafting as a central adaptive process in technologically mediated work environments rather than as a peripheral response to organizational change.

The review also reinforces the relevance of cognitive appraisal theory for understanding employee adaptation to workplace AI. The synthesized evidence indicates that AI-related perceptions and cognitions are associated with job crafting less through stable direct relationships than through employees’ interpretations of AI as a challenge or a threat. This underscores appraisal as a key interpretive process through which technological conditions become linked to behavioral adjustment.

A further contribution lies in the conceptual clarification of the broad label of AI awareness. Rather than reflecting a single unified construct, the included studies examined a heterogeneous set of AI-related perceptions and cognitions, including exposure-based, evaluative, and capability-related indicators. Recognizing this heterogeneity helps explain the inconsistency of direct associations across studies and supports greater conceptual precision in future theorizing.

The review additionally refines current understandings of employee adaptation in AI-enabled work contexts by showing that job crafting should not be conceptualized exclusively as constructive or growth-oriented. Alongside approach-oriented crafting, the evidence also points to defensive and avoidance-oriented forms of adjustment. A more complete theoretical account of AI-related adaptation should therefore incorporate both developmental and protective pathways of employee agency.

### 4.5 Practical implications

The findings of this review carry practical implications for organizations implementing AI in the workplace. Most importantly, the evidence suggests that employee responses to AI depend not only on the introduction of the technology itself, but also on how it is interpreted in relation to work demands, professional identity, and perceived control. AI implementation should therefore be understood not merely as a technical process, but as a psychologically consequential form of organizational change.

For managers and organizations, this highlights the importance of transparent communication, developmental support, and participatory implementation practices. Clear explanations of why AI is being introduced, how it will affect work roles, and what forms of support will be available may help reduce uncertainty and weaken threat-oriented appraisals. At the same time, training should not be limited to technical skill acquisition. AI-related knowledge may also function as a psychological resource by strengthening employee confidence and supporting challenge-oriented interpretations of AI. Work systems that preserve role clarity, meaningful autonomy, and opportunities for collaborative human–AI interaction may therefore be more likely to foster adaptive forms of job crafting than systems experienced primarily as surveillance, replacement, or rigid control.

For employees, the findings suggest that successful adaptation to AI may require more than technical upskilling alone. Access to social support, sensemaking opportunities, and resources that strengthen perceived efficacy may help employees reinterpret AI-related change as manageable and potentially beneficial. In this sense, effective adaptation involves not only learning to use AI tools, but also renegotiating work roles and maintaining a sense of professional value in changing work environments.

## 5 Conclusion

AI-related perceptions and cognitions in the workplace do not produce uniform patterns of employee adaptation. Instead, employees’ responses to AI are shaped by how its implications are cognitively appraised within specific individual and organizational contexts. When AI is interpreted as a challenge, employees are more likely to engage in approach-oriented job crafting, including proactive task modification, skill development, and role expansion. When AI is appraised as a threat, the AI-related constructs examined in the reviewed studies are more often associated with avoidance-oriented forms of job crafting and protective forms of work regulation. Overall, the evidence suggests that AI awareness should not be treated as a single homogeneous construct but rather as an umbrella term encompassing multiple AI-related perceptions and cognitions whose behavioral consequences depend on appraisal processes and contextual conditions.

These findings further suggest that observed behavioral patterns surrounding AI in organizations cannot be understood solely in terms of technological capabilities or implementation intensity. Rather, they appear to be closely related to employees’ interpretive processes and to the resources available to support adaptive sensemaking. Individual factors such as AI-related knowledge and a positive stress mindset, as well as organizational conditions including supportive leadership and ethical organizational signals, appear to influence whether AI-related perceptions are associated with more adaptive or more defensive work behaviors.

Viewed through a cognitive appraisal lens, artificial intelligence does not diminish employee agency in work design but renders it contingent and context-dependent. Job crafting in AI-enabled work systems reflects not only proactive self-initiated change but also employees’ ongoing efforts to preserve meaning, control, and professional identity under conditions of technological uncertainty. Recognizing these appraisal-based dynamics highlights the importance of designing organizational environments that support constructive interpretations of AI and foster more sustainable and effective human–AI collaboration.

## Data Availability

The original contributions presented in the study are included in the article/supplementary material, further inquiries can be directed to the corresponding author.
